# Undifferentiated Pleomorphic Sarcoma of the Distal Thigh: A Case Report

**DOI:** 10.7759/cureus.58685

**Published:** 2024-04-21

**Authors:** Osama S AlShaya, Saud R Alanazi, Faisal M Alshaghathirah

**Affiliations:** 1 Reconstructive Orthopedic Department, King Fahad Medical City, Riyadh, SAU; 2 Orthopedic Department, King Fahad Medical City, Riyadh, SAU; 3 College of Medicine, Imam Mohammad Ibn Saud Islamic University, Riyadh, SAU

**Keywords:** thigh, sarcoma, radiotherapy, pleomorphic, orthopedic

## Abstract

The undifferentiated pleomorphic sarcoma (UPS) is a rare malignant tumor of mesenchymal origin. Poorly differentiated tumor cells, which might take the form of giant cells, histiocytes, or spindle-shaped cells, make up the UPS variant of sarcomas. If soft tissue tumors enlarge and turn malignant, they may become an issue. Sarcoma is diagnosed by several tests, such as a physical examination, MRI, CT scan, or ultrasound. A biopsy yields information regarding the grade and subtype of the sarcoma and is required for a clear diagnosis. Chemotherapy, radiation therapy, and broad-margin excision are the standard treatments for cancers of the bone.

UPS often appears in people between 50 and 70 years old. Yet, here we report a 40-year-old male diagnosed with UPS. Our goal is to discuss how unique our case is in comparison to others, as well as the available diagnostic and therapeutic alternatives in such cases.

## Introduction

Undifferentiated pleomorphic sarcoma (UPS) is a rare malignant tumor of mesenchymal origin, previously known as malignant fibrous histiocytoma. The tumor's presumed source is mesenchymal stem cells. It can affect soft tissues, bones, and the retroperitoneum and spread to many organs [1]. Poorly differentiated tumor cells, which might take the form of giant cells, histiocytes, or spindle-shaped cells, make up the UPS variant of sarcomas [2]

UPS was the second most prevalent soft-tissue sarcomas (STS) after leiomyosarcoma, regardless of the underlying tumor site, accounting for 17.1% of 26,758 cases, as per the Surveillance, Epidemiology, and End Results (SEER) program. Male incidence rates were significantly higher than female incidence rates, with White males being affected more frequently than Black males. Additionally, the incidence grew linearly with increasing age [3]

Due to the non-specific histology of this disorder's tumors, UPS is a diagnosis of elimination. Microscopically, UPS tumor cells are pleomorphic and undifferentiated. Since UPS tumor cells express a particular set of proteins but not those of other undifferentiated and pleomorphic tumors, UPS's diagnosis is frequently predicated on the detection of those proteins [4]. UPS often appears in people between 50 and 70 years. Yet, here we report a 40-year-old male diagnosed with UPS with the goal of discussing how unique our case is in comparison to others, as well as the available diagnostic and therapeutic alternatives in such cases.

## Case presentation

A 40-year-old male who is otherwise medically healthy presented initially in March 2023, complaining of swelling in his right distal thigh, which has since become larger and been accompanied by slight pain. There was night sweating and fever, but no weight loss or signs of tiredness. There was no medical or surgical history of significance or known allergies. In addition, there was no history of chest pain, shortness of breath, numbness, abdominal pain, or cough. He has been a smoker for 15 years. The pain and the swelling became more progressive over a period of five months, and then he decided to seek medical advice.

**Figure 1:**
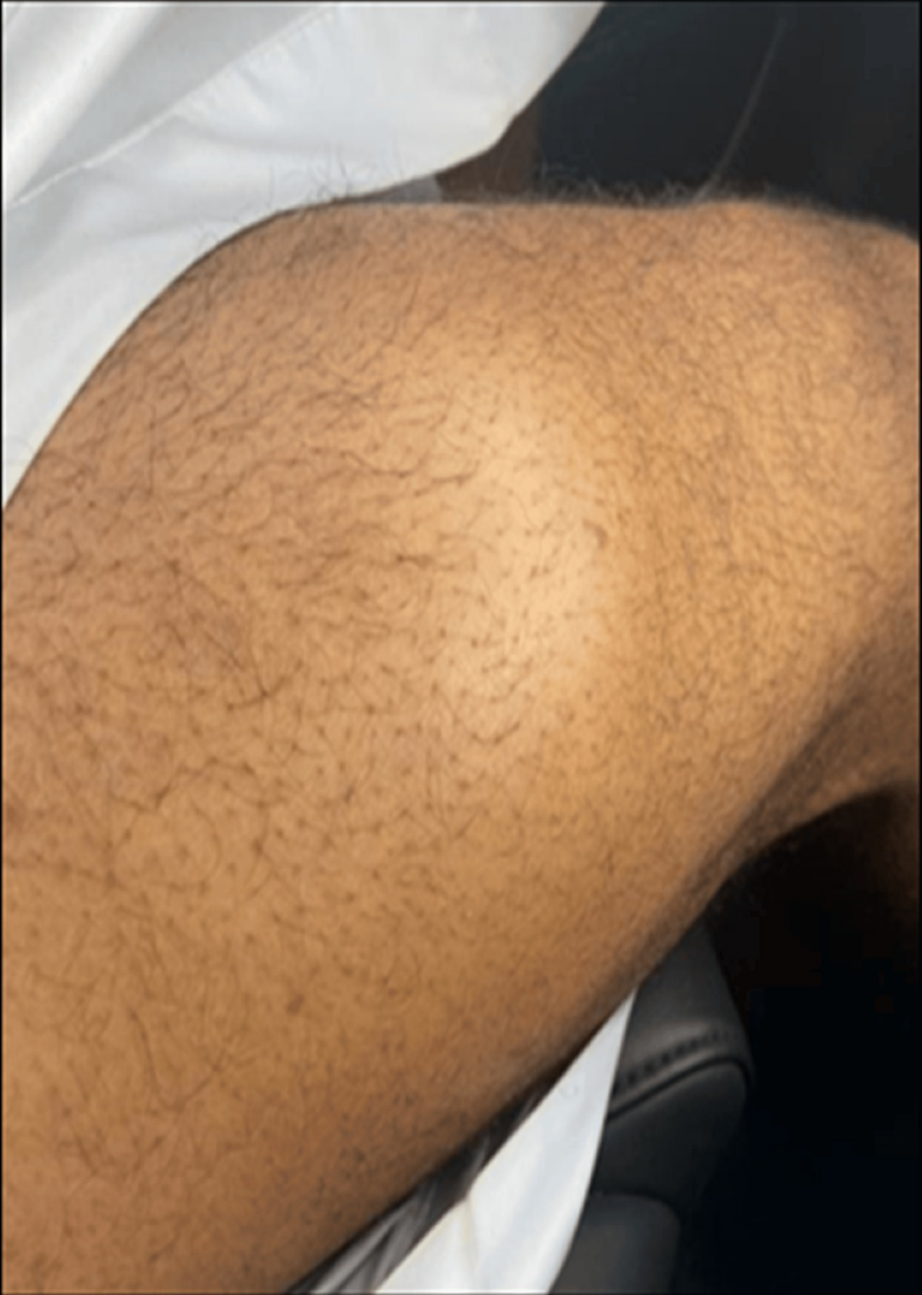
Clinical picture of the lesion (involved distal anterior-lateral thigh) A globular swelling on the right distal anterior-lateral aspect of the thigh around 12 x 12 cm.

During a complete physical examination, the patient was able to walk normally. There was a globular swelling on the right distal anterior-lateral aspect of the thigh around 12 x 12 cm. It was not tender or mobile, and the temperature over the swelling was normal. There were no changes in sensation or deep tendon reflexes. Knee flexion, extension, adduction, and abduction were all normal (Figure 1).

**Figure 2:**
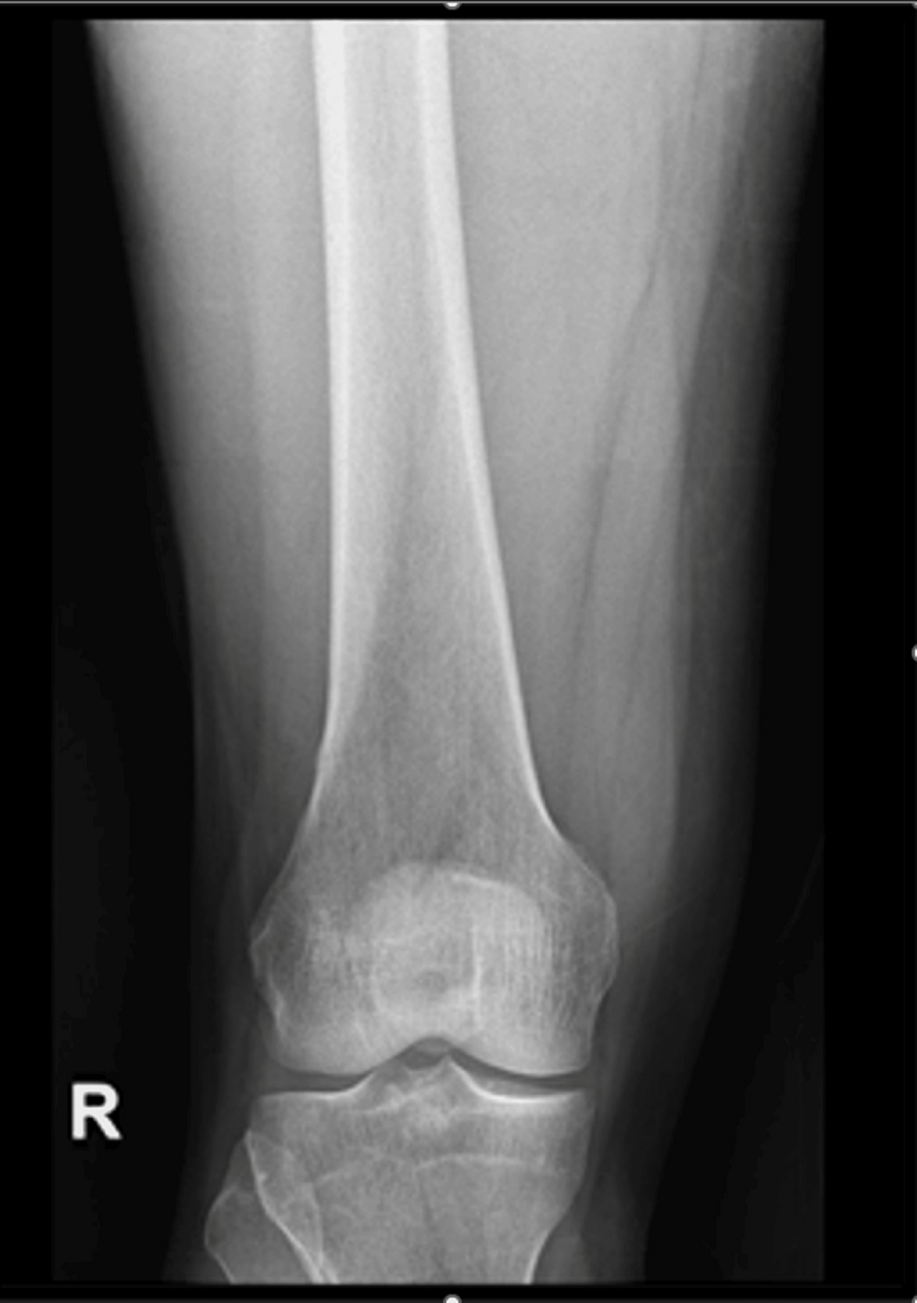
Anterior-posterior X-ray view Normal X-ray of the femur bone

**Figure 3:**
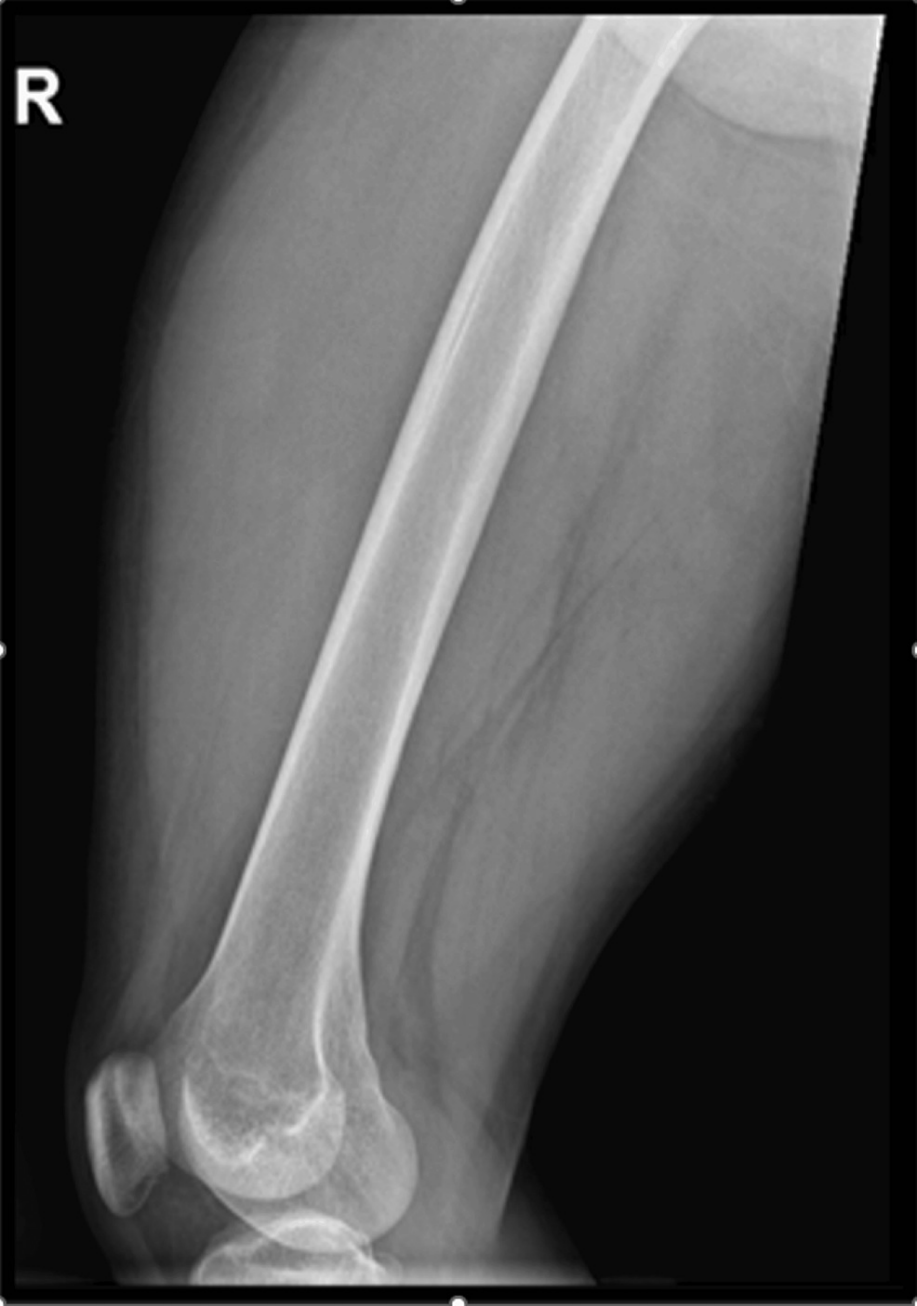
Lateral X-ray view Normal X-ray of the femur bone

A complete workup was done for the patient, including imaging (Figures 2, 3).

**Figure 4:**
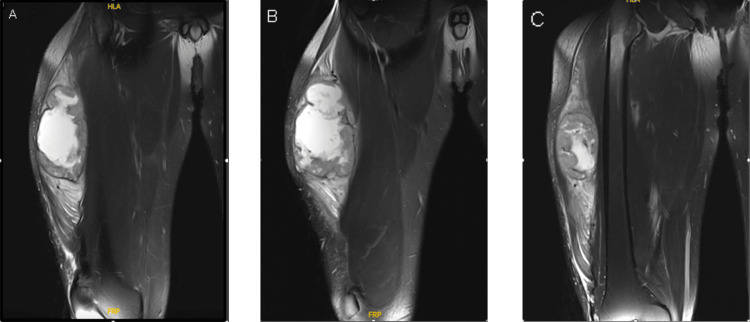
MRI T2 coronal cut (A) most anterior cut; (B) middle cut; (C) posterior cut This lesion measured about 8.7 x 8 x 12 cm

A tissue sample was taken before presentation in another facility. The right thigh magnetic resonance imaging (MRI) done in March 2023 showed a right ventrolateral swelling with heterogeneous cystic and solid components, mainly in the vastus lateralis, which showed a low signal on T1 and a high signal in T2 with non-fatty component and central necrotic area with heterogeneous post-contrast enhancement. This lesion measured about 8.7 x 8 x 12 cm, compared to a previous MRI done in December 2022 in another facility, and the lesion measured about 5 x 7.2 cm. There is diffusion restriction. There was regional muscle edema with pos-contrast enhancement. There were no other lesions detected. The femoral bone was unremarkable, with no lymphadenopathy. The neurovascular bundle was intact (Figure 4).

Computed tomography (CT) of the chest was done in our facility as part of the workup, and no definite metastatic disease was observed in the chest. CT of the abdomen and pelvis showed a non-specific prominent right iliac group of lymph nodes for follow-up. Otherwise, there was no evidence of abdominopelvic metastasis.

A bone scan was done, and it showed a soft tissue mass in the right thigh, likely attributed to the known disease. There was also a small faint radiotracer uptake at the right 10th posterior rib without an evident underlying lesion. Otherwise, there was no scintigraphic evidence of active bone metastasis. A tissue biopsy under ultrasound guidance was performed in another facility and revealed high-grade undifferentiated pleomorphic sarcoma.

After all the investigations and determining the final diagnosis, the plan was to start neoadjuvant radiotherapy followed by surgical resection. In April 2023, he started radiotherapy. The course was 5000cGy in 25 fractions, which was completed in May 2023.

After finishing the radiotherapy course, the MRI was repeated and showed that there was an interval-dimensional and morphological progression of the right soft tissue mass involving the anterior muscle compartment. The mass measured 16 cm in the craniocaudal dimension and demonstrated significant intra-lesional necrotic and hemorrhagic changes with the persistence of peri-lesional soft tissue edema. There was no involvement of the bones, no lymphadenopathy, and no involvement of the neurovascular bundle.

**Figure 5:**
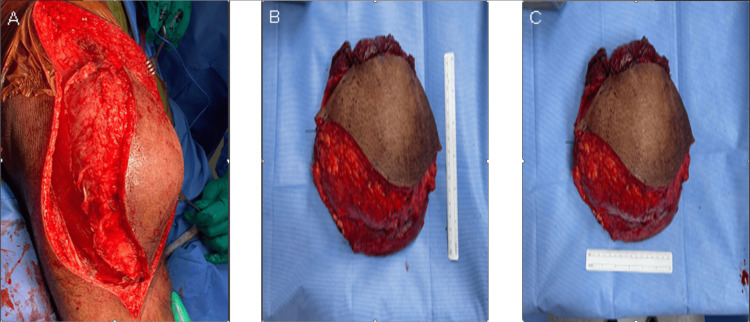
Operative images (A) intraoperative; (B) postoperative; (C) postoperative The tumor size was 15 cm in the greatest dimension.

In June 2023, the patient underwent marginal soft tissue mass resection, open biopsy, and primary closure. The histopathology revealed treated UPS with residual focal scattered pleomorphic cells (3%). Treatment effect necrosis presents 95%. The tumor size was 15 cm in the greatest dimension. All margins were negative for tumor involvement (the closest margin was a lateral margin of 0.5 cm from the mass, with treatment effect) (Figure 5).

After discussing the options of chemotherapy and observation with the patient, he chose observation. The patient started physical therapy. He modified his activities and became independent in activities of daily living and walking outdoors. He is improving and recovering during the follow-up. In September and again in December 2023, follow-up MRIs were performed and revealed no concerning enhancing nodule.

## Discussion

Undifferentiated pleomorphic sarcomas/malignant fibrous histiocytomas are high-grade pleomorphic malignant tumors that lack particular immunohistochemistry markers for a specific differentiation lineage [5]. As in our case, the tumor originated in the thigh, which is a common site of UPS (the most common sites for UPS are the proximal femur, proximal humerus, and retroperitoneum). It is more likely for this tumor to appear later in life, especially in the seventh decade [6]. One of the aspects that sets our situation apart from others is that the patient was relatively young when compared to the typical age range.

The diagnosis of sarcoma is made through a number of tests. First, a physical examination determines the condition's medical signs and symptoms. Any masses may be found with an ultrasound. The precise size, shape, and degree of the sarcoma's involvement with the surrounding tissue is often assessed using an MRI (regarded as the gold standard) or a CT scan. Sarcoma must be definitively diagnosed with a biopsy. The biopsy-taken tissue reveals details regarding the grade and subtype of the sarcoma. The grade of the patient's sarcoma's histology significantly impacts the patient's prognosis [7]. Sarcoma diagnoses are frequently delayed. Due to their rarity, they usually elude a doctor's differential diagnoses. Additionally, because the mass is typically painless, patients frequently delay seeking urgent treatment [8]. Just like in our case, the patient presented with vague symptoms, making it challenging to identify the disease quickly.

The United Kingdom Department of Health has established guidelines for the urgent referral of any patient with a soft tissue lesion, including the following: mass greater than 5 cm (golf ball size), painful lump, growing lump, a lump of any size deep to the muscle fascia, or any lump that returns after excision [9]. The three different treatment options include excision with wide margins. The remainder of the treatment, which may include radiation therapy and chemotherapy, is dependent on the tumor's grade, local invasion, and metastasis. In our situation, for example, we used radiotherapy and wide-margin excision.

The involvement of the lymph nodes must be thoroughly evaluated, not just for dissection but also to ensure that no spread has occurred [6]. Soft tissue sarcomas of the extremities must be entirely eradicated from the body in order to be cured [6,10]. Thankfully, there was no metastasis in our situation.

Wide resection is the most popular method for eliminating tumors; it entails removing a sizeable portion of the surrounding healthy tissues [7,11,12]

A positive margin was shown to be the most significant predictive indicator for identifying patients who were likely to experience a recurrence. It's critical to identify and assess any expanding tumor right away because sarcomas have a significantly better prognosis with early detection and smaller size. Sarcomas are uncommon. However, they should not be disregarded when examining any soft tissue mass. It is crucial to promptly report any suspicious cases to the proper management facilities and to pursue vigorous treatment to avoid consequences [8]

## Conclusions

Though extremely uncommon soft tissue neoplasms, undifferentiated pleomorphic sarcomas should not be ruled out as a differential when a patient appears with a growing mass, particularly if the lump is in the extremities. With aggressive sarcomas, it is best to prevent delay in diagnosis and treatment because the chance of metastasis rises with tumor size.

The best tool for diagnosing pleomorphic sarcoma is MRI. Even after aggressive therapy, the risk of relapse is high, especially within the first few years. That is why regular MRI imaging is necessary.
